# Chorologie des Tabanidae (Diptera) dans la réserve de biosphère Ipassa-Makokou (Gabon) en saison des pluies

**DOI:** 10.1051/parasite/2012192165

**Published:** 2012-05-15

**Authors:** J.F. Mavoungou, B.K. Makanga, G. Acapovi-Yao, M. Desquesnes, B. M’batchi

**Affiliations:** 1 Institut de Recherches en Écologie Tropicale (IRET) BP 13354 Libreville Gabon; 2 Université des Sciences et Techniques de Masuku (USTM) BP941 Franceville Gabon; 3 Université Cheikh Anta Diop, Faculté des Sciences et Techniques BP 5005 Dakar-Fann Sénégal; 4 Université d’Abidjan, Cocody, UFR Biosciences 22 BP 582 Abidjan 22 Côte d’Ivoire; 5 Centre de Coopération Internationale en Recherche Agronomique pour le Développement (CIRAD), UMR Intertryp 34398 Montpellier France

**Keywords:** Tabanidae, parc, réserve, piège Vavoua, biotope, saison des pluies, Gabon, Tabanidae, park, reserve, Vavoua traps, biotope, rainy season, Gabon

## Abstract

L’abondance et la diversité spécifique des tabanidés ont été évaluées par des captures d’insectes à l’aide de pièges Vavoua durant la saison des pluies, du 4 octobre au 30 novembre 2009, dans trois types de biotope : forêt primaire, forêt secondaire et villages, dans la réserve de biosphère Ipassa-IRET Makokou au Gabon. Huit espèces de tabanidés appartenant à trois genres ont été identifiées sur un total de 402 spécimens capturés. Les espèces de tabanidés numériquement les plus abondantes ont été : *Tabanus secedens* Walker, 1854 (55,2 %), *Tabanus obscurehirtus* Ricardo, 1908 (13,9 %), *Chrysops dimidiatus* Wulp, 1885 (11,2 %) et *Chrysops silaceus* Austen, 1907 (10,7 %). Les espèces les moins abondantes ont été *Tabanus par* Walker, 1854 (3,2 %), *Tabanus besti arbucklei* Austen, 1912 (3 %), *Tabanus marmorosus congoicola* Bequaert, 1930 (1 %) et *Ancala fasciata fasciata* (Fabricius, 1775) (0,5 %). Des spécimens des genres *Tabanus* et *Chrysops* n’ont pu être déterminés, représentant respectivement des taux de 0,7 % et 0,5 % des insectes capturés. La plus forte proportion de tabanidés a été capturée en forêt secondaire (75,1 %) et la plus faible en forêt primaire (4,5 %).

## Abstract (en)

The abundance and species diversity of tabanids were evaluated by trapping of insects using Vavoua traps, during the rainy season, from October 4 to November 30, 2009, in three different habitats: primary forest, secondary forest and village, in the biosphere reserve Ipassa-IRET Makokou in Gabon. Eight species belonging to three genera of tabanids have been identified for a total of 402 specimens caught. The tabanid species numerically the most abundant were: *Tabanus secedens* Walker, 1854 (55.2%), *Tabanus obscurehirtus* Ricardo, 1908 (13.9%), *Chrysops dimidiatus* Wulp, 1885 (11.2%) and *Chrysops silaceus* Austen, 1907 (10,7%). The less abundant species were *Tabanus par* Walker, 1854 (3.2%), *Tabanus besti arbucklei* Austen, 1912 (3%), *Tabanus marmorosus congoicola* Bequaert, 1930 (1%) and *Ancala fasciata fasciata* (Fabricius, 1775) (0.5%). Specimens of the genera *Tabanus* and *Chrysops* could not be identified, these insects represented respectively 0.7% and 0.5% of the insects trapped. The highest proportion of tabanids was trapped in secondary forest (75.1%) and the lower in primary forest (4.5%).

La connaissance des insectes hématophages présente beaucoup d’importance en médecine humaine et vétérinaire (Leclercq, 1967). Les tabanidés, particulièrement ceux appartenant au genre *Tabanus* et aux genres voisins (*Atylotus* et *Ancala*), de par leur comportement alimentaire et la nature de leurs pièces buccales, seraient les vecteurs mécaniques les plus efficaces avant les stomoxes du genre *Stomoxys* (Oldroyd, 1954; Zumpt, 1973). Les tabanidés sont ainsi des vecteurs potentiels dans la transmission mécanique d’agents pathogènes (virus, bactéries, parasites), notamment des trypanosomes (Rodhain & Perez, 1985; Foil, 1989). Quelques espèces de tabanidés, appartenant au genre *Chrysops*, sont les vecteurs de la filariose à *Loa loa* (Van Hoegaerden *et al.*, 1987; Touré *et al*., 1998, 1999).

Au Gabon, les connaissances sur ces insectes hématophages sont peu documentées (Mavoungou *et al.*, 2008; Kohagne *et al.*, 2010, 2011). La faune des tabanidés dans les parcs nationaux du Gabon (Vande weghe, 2006) demeure jusqu’à présent assez mal connue (Makanga, 2009). Pourtant, ces insectes hématophages peuvent représenter un risque pour les populations humaines et animales par leur nuisance directe, mais également par leur rôle de vecteurs. Par ailleurs, la meilleure valorisation des parcs nationaux du Gabon en termes d’écotourisme passe nécessairement par la connaissance de la distribution et de l’abondance des différentes espèces d’insectes vecteurs de pathogènes. Cette connaissance est utile à l’élaboration de stratégies de contrôle de ces insectes. Pour recueillir des données sur la distribution des tabanidés, une enquête entomologique de nature transversale a été réalisée dans trois biotopes différents qui caractérisent la réserve de biosphère incluse dans le Parc National de l’Ivindo.

L’objectif de cette enquête était de déterminer les densités apparentes des tabanidés dans les trois principaux biotopes : forêt primaire, forêt secondaire, village.

## Matériel et Méthodes

### Milieu d’étude

**Figure 1.**
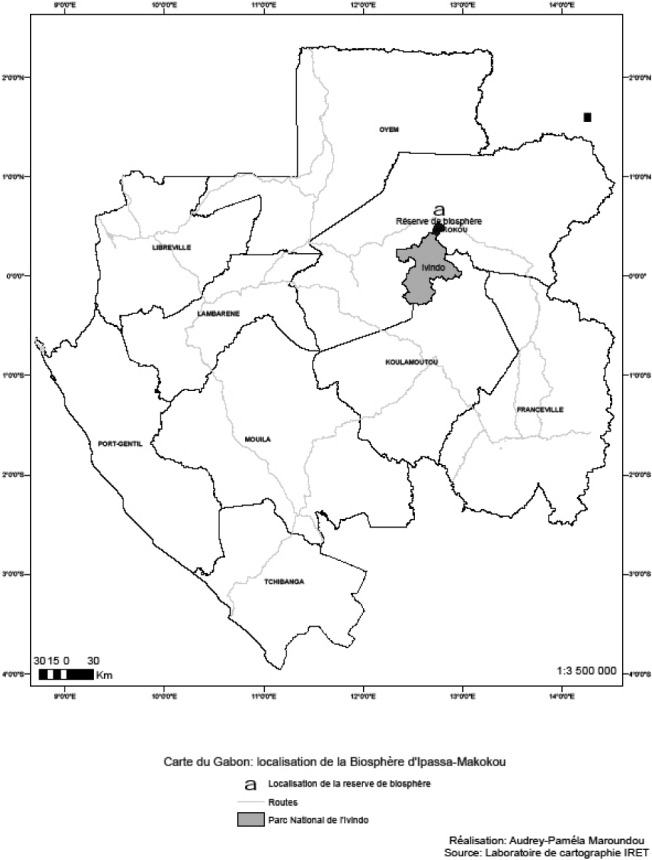
Représentation du site d’étude.

L’étude a été réalisée dans la région de Makokou qui se situe dans la Province de l’Ogooué-Ivindo, dans le Nord-Est du Gabon, à 620 km environ de Libreville (figure 1). Les sessions de piégeage se sont déroulées dans les environs de la station de recherche de l’Institut de Recherches en Écologie Tropicale (IRET) d’Ipassa. Cette dernière est située en zone forestière à 12 km de la ville de Makokou (0° 23’ – 0° 33’ N; 12° 42’ – 12° 49’E) et à 500 m d’altitude (Wilks, 1990).

Le climat de la région est de type équatorial humide caractérisé par la double alternance des saisons sèches et pluvieuses. L’année est divisée en quatre saisons plus ou moins également réparties. On distingue :

- la petite saison sèche qui s’étend de mi-décembre à mi-mars. Elle se caractérise par un ciel souvent dégagé et quelques pluies épisodiques. En fin de saison, l’humidité chute parfois fortement;
- la petite saison des pluies qui s’étale de mi-mars à mi-juin. Les précipitations, parfois orageuses augmentent; l’insolation atteint son maximum;
- la grande saison sèche entre mi-juin et mi-septembre. L’effet négatif de la forte diminution des précipitations sur la végétation est tempéré par une nébulosité importante quasi permanente tout le long de la saison sèche. L’insolation chute, les températures sont les plus basses de l’année, l’évaporation diurne est minimale, l’humidité reste élevée;
- la grande saison des pluies à partir de mi-septembre. Les orages sont fréquents, surtout en début de saison, et parfois accompagnés de tornades.

La pluviométrie moyenne annuelle est d’environ 1 600 à 1 800 mm. La température moyenne est proche de 24 °C. Les amplitudes thermiques annuelles et journalières sont faibles. L’enquête a été réalisée entre le 4 octobre et le 30 novembre 2009, pendant la grande saison des pluies. La faune forestière gabonaise est riche et diversifiée. La région de Makokou en détient une part importante avec une faune parmi la mieux répertoriée du Gabon. Elle est riche, entre autres, de 128 espèces de mammifères, 424 espèces d’oiseaux, 65 espèces de reptiles et 47 espèces d’amphibiens.

### Capture des tabanidés

#### • Choix du piège

Plusieurs types de pièges pouvaient être utilisés dans cette étude, notamment le piège Vavoua qui permet la capture des stomoxes et des tabanidés, en particulier les *Chrysops*, vecteurs de la loase, et le piège Nzi qui est particulièrement efficace pour la capture des tabanidés. Le choix de n’utiliser que le piège Vavoua dans cette étude a été déterminé par le budget. En effet, sous contrainte de coût à ne pas dépasser, le dispositif d’échantillonnage optimal a été celui basé sur le seul piège Vavoua (moins cher) plutôt que sur le piège N’Zi (plus efficace à l’unité mais plus cher).

#### • Piégeage

Le piégeage a consisté en un réseau de 12 pièges, placés aux mêmes points identifiés au préalable le long d’un transect d’environ 9 km, suivant le gradient d’anthropisation allant de la forêt primaire (milieu non anthropisé), passant par la forêt secondaire (milieu partiellement anthropisé) et allant jusqu’au village Loaloa (milieu fortement anthropisé). Le piégeage s’est déroulé sur une durée de deux mois, avec une session de piégeage par semaine. L’effort de piégeage dans la semaine a été de quatre jours consécutifs. Les 12 pièges ont été répartis dans les trois milieux d’étude, à raison de quatre pièges par milieu. Au total, le nombre de piège-jours s’est donc élevé à :

12 pièges × 4 jours par semaine × 8 semaines = 384 pièges-jours

Les pièges Vavoua (Laveissière & Grebaut, 1990) ont été activés le matin avant 7 h et relevés le soir après 18 h pendant quatre jours consécutifs par semaine. Lors de la relève des pièges, les cages de capture ont été étiquetées avec le numéro du piège et la date, et ramenées au laboratoire. Elles ont été ensuite placées dans un congélateur durant 15 minutes pour tuer les insectes avant de les stocker dans des flacons contenant de l’éthanol à 95°.

#### • Identification des tabanidés

Au laboratoire, les insectes capturés ont été triés en séparant les tabanidés des autres Diptères. Les identifications des différentes espèces de tabanidés ont été faites sous une loupe binoculaire à l’aide des clés publiées par Oldroyd (1952, 1954, 1957, 1973) et en s’appuyant sur la collection de référence des tabanidés de l’Institut Fondamental d’Afrique Noire (IFAN) à l’Université Cheikh Anta Diop à Dakar.

#### • Analyse des données

L’indice de diversité de Margalef a été calculé pour évaluer la diversité des tabanidés dans la réserve de biosphère. Cet indice se calcule à l’aide de la formule suivante : D = (S - 1)/log N, où “S” est le nombre d’espèces et “N” le nombre total d’individus récoltés (Legendre & Legendre, 1979). Le test du χ^2^ a été effectué pour comparer la distribution des différentes espèces en fonction du biotope.

## Résultats

### Composition des populations de tabanidés capturées

Au total, 402 tabanidés ont été récoltés, en 384 piège-jours, soit une densité apparente par piège (DAP) à peine supérieure à 1 (DAP = 1,05). Sur ce total, 397 spécimens ont pu être identifiés, appartenant à trois genres et huit espèces. Cinq spécimens n’ont pu être identifiés au-delà du genre.

#### • Composition par genre

**Figure 2.**
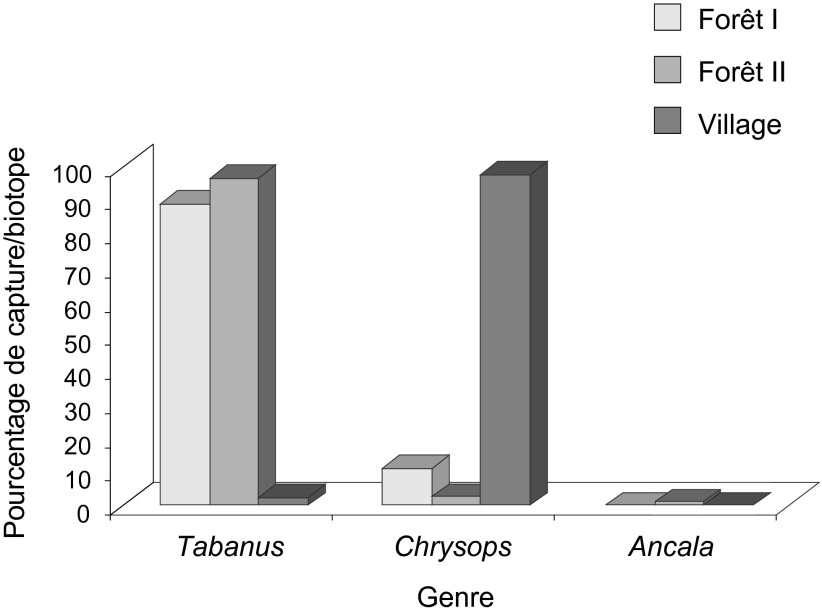
Répartition des genres de tabanidés capturés en fonction des biotopes prospectés.

Sur l’ensemble des biotopes, le genre *Tabanus* a été le plus abondant (77,1%), suivi du genre *Chrysops* (22,4%) et du genre *Ancala* qui a été très faiblement représenté avec (0,5%). En forêt primaire et secondaire, le genre *Tabanus* a été le plus représenté avec des taux respectifs de 88,9 et 96,7%. Tandis qu’au village, le genre *Chrysops* a été le plus représenté avec un taux de 97,6% (figure 2).

#### • Composition par espèce

**Figure 3.**
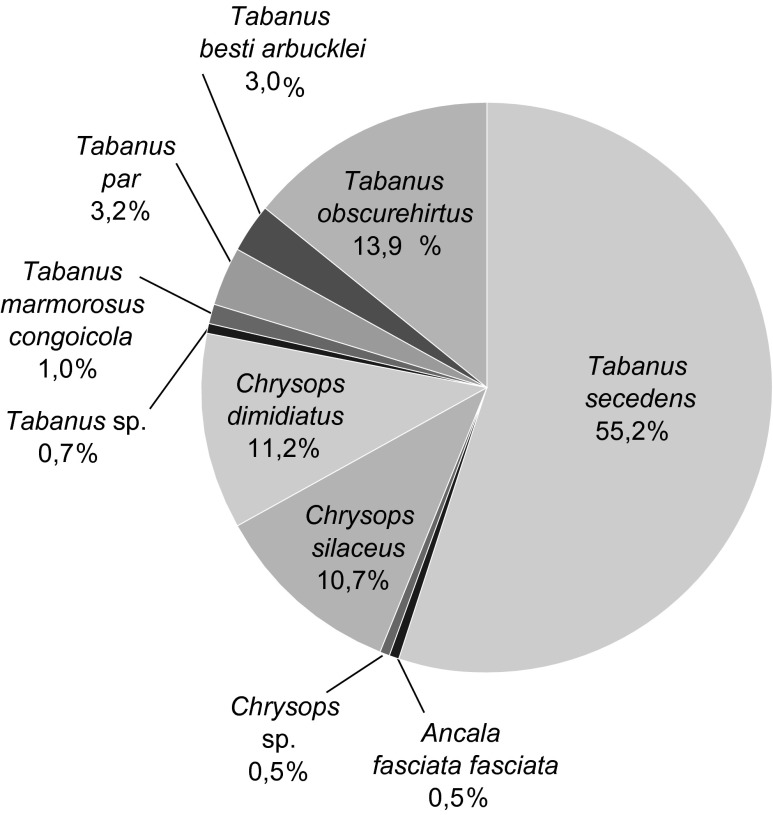
Répartition spécifique des espèces de tabanidés capturés.

Le genre *Tabanus* comprenait cinq espèces : *Tabanus secedens* (55,2%), *Tabanus obscurehirtus* (13,9%), *Tabanus par* (3,2%), *Tabanus besti arbucklei* (3%) et *Tabanus marmorosus congoicola* (1%) (figure 3). Trois spécimens de *Tabanus*, appartenant très probablement à la même espèce, n’ont pu être identifiés au-delà du genre.

Le genre *Chrysops* comprenait deux espèces : *Chrysops dimidiatus* (11,2%) et *Chrysops silaceus* (10,7%). Deux spécimens de *Chrysops* appartenant à la même espèce n’ont pu être identifiés au-delà du genre.

Le genre *Ancala* comprenait une seule espèce : *Ancala fasciata fasciata* (0,5%).

### Abondance relative des tabanidés en fonction des biotopes étudiés

**Figure 4.**
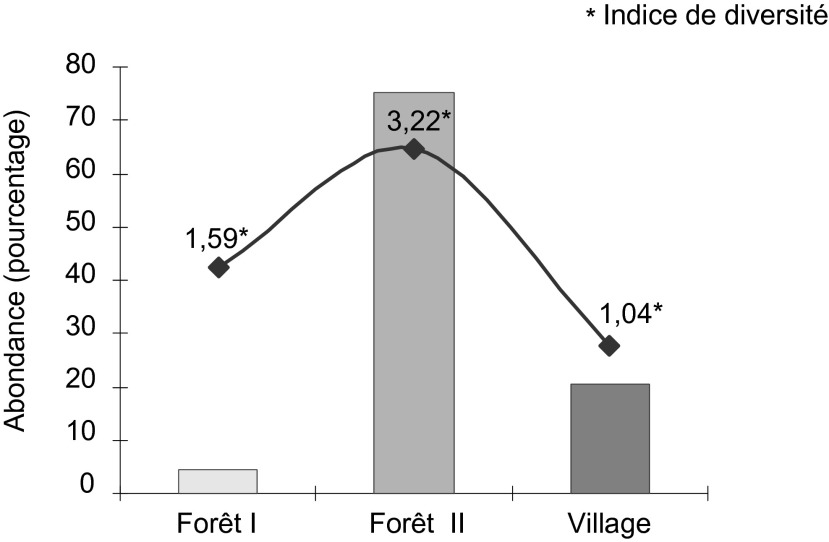
Abondance et diversité des espèces de tabanidés capturées selon le biotope.

Le maximum de captures a été observé en forêt secondaire (75,1%), suivi des captures au village (20,4%) et le minimum a été noté en forêt primaire (4,5%) (figure 4). La diversité du peuplement des tabanidés dans les différents biotopes a été évaluée par l’indice de Margalef. Si l’on tient compte des deux espèces qui n’ont pas pu être identifiées en forêt secondaire, les indices de diversité ont été de 3,22 en forêt secondaire; 1,59 en forêt primaire et 1,04 au village.

En forêt primaire, trois espèces ont été capturées : *T. secedens* a été le mieux représenté avec 55,6%, suivi de *T. obscurehirtus* (33,3%), puis de *C. dimidiatus* (11,1%). Les autres espèces de tabanidés ont été absentes dans les captures.

**Figure 5.**
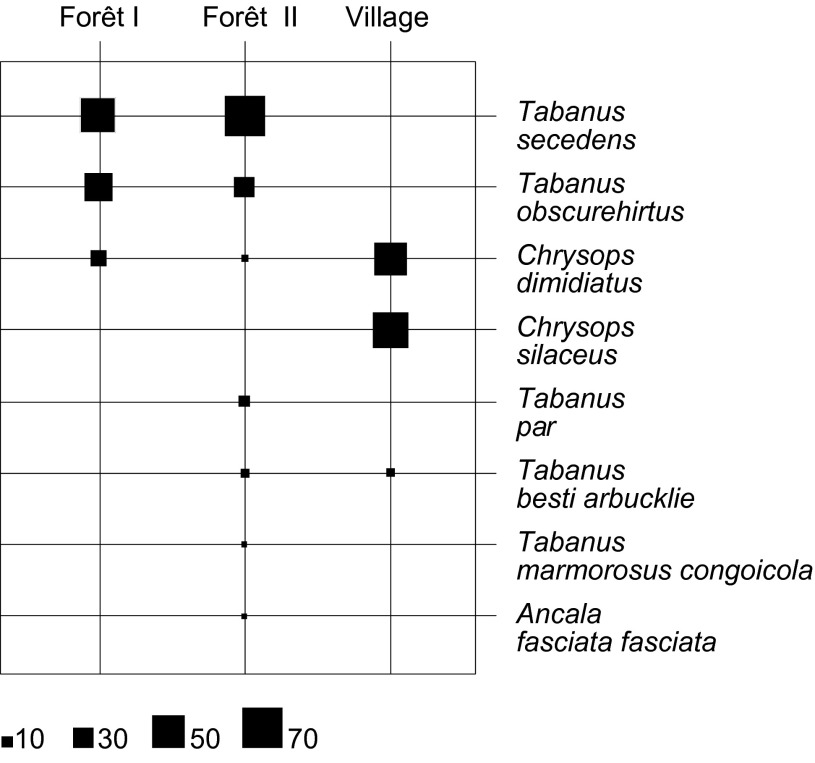
Distribution des différentes espèces de tabanidés en fonction du biotope.

En forêt secondaire, une forte richesse spécifique caractérisée par la présence de neuf espèces a été observée : *T. secedens* (70,2%), *T. obscurehirtus* (16,6%), *T. par* (4,3%), *T. besti arbucklei* (3,3%), *T. marmorosus congoicola* (1,3%), *C. dimidiatus* (2%), *An. fasciata fasciata* (0,7%), ainsi que *Tabanus* sp. (trois spécimens) et *Chrysops* sp. (deux spécimens) qui n’ont pu être identifiés au niveau de l’espèce du fait de l’absence de spécimens correspondants dans les collections de références. À noter que les trois espèces capturées en forêt primaire sont présentes en forêt secondaire (figure 5).

Dans le village, milieu anthropisé, trois espèces ont été capturées : *C. silaceus* (52,4%), *C. dimidiatus* (45,1%) et *T. besti arbucklei* (2,4%). *C. silaceus* a présenté une forte abondance au village mais était absent des deux autres biotopes. *C. dimidiatus* était présent dans les trois biotopes. *T. besti arbucklei* est présent à la fois au village et en forêt secondaire, mais avec une faible abondance.

Le résultat du test du χ^2^ a révélé une différence hautement significative au seuil de 0,001 p. 100 dans la distribution des différentes espèces selon le biotope (χ^2^ = 359; ddl = 18; p < 0,001).

## Discussion

Les résultats obtenus dans cette étude constituent des données préliminaires sur les tabanidés de la réserve de biosphère à Makokou, au Gabon. Les faibles captures enregistrées (DAP = 1,05) pourraient s’expliquer par l’utilisation du piège Vavoua. En effet, des travaux menés par Mihok (2002) et Acapovi (2005) ont montré une efficacité supérieure du piège N’Zi pour la capture des tabanidés et du piège Vavoua pour la capture des stomoxes. Le piège Vavoua seul a été utilisé dans cette étude pour des raisons budgétaires.

Au total, huit espèces ont été identifiées, ce qui représente 10% des espèces connues en Afrique centrale (Taufflieb & Finelle, 1956). Cette faible richesse spécifique observée dans cette région du Gabon pourrait s’expliquer par le fait qu’un grand nombre de sites n’ont pas été visités et que seulement 12 points de captures ont été explorés, pendant une période relativement courte à une saison unique, et à l’aide d’un seul type de piège.

Des études précédentes ont montré que l’association de plusieurs types de pièges (N’Zi, Grand Tetra, Petit Tetra) et l’ajout d’un attractif olfactif, l’octénol, augmentaient significativement les captures des tabanidés (Djiteye, 1992; Amsler & Filledier, 1994).

La période de capture semble être une des causes des faibles effectifs enregistrés. En effet, la période d’octobre à novembre, grande saison des pluies, est caractérisée par de nombreux orages accompagnés de fortes précipitations qui sont néfastes aux populations adultes, du fait de leur violence, et qui peuvent engendrer une dormance temporaire des populations larvaires lorsque leur milieu de développement est inondé (Raymond, 1988; Desquesnes *et al.*, 2005). Lorsque les précipitations sont plus faibles, elles ne sont pas favorables à l’activité des tabanidés, ainsi, en Mauritanie, Dia *et al.* (1998) obtiennent un maximum de captures pendant la saison des pluies entre octobre et novembre.

Les tabanidés étant des insectes saisonniers, selon les milieux et les espèces, les pics d’abondance peuvent survenir en saison sèche ou humide. Des travaux conduits par Desquesnes *et al.* (2005) à Lahirasso au Burkina Faso ont montré que les tabanidés sont très abondants à partir du mois de novembre, ce qui correspond à la saison sèche. Dans certaines zones telles qu’en Guyane française, certaines espèces se rencontrent tout au long de l’année, mais la plupart ont un pic d’abondance marqué en saison sèche, particulièrement vers la fin, en novembre, dès les premières pluies (Raymond, 1988). L’activité des tabanidés diminue brutalement, pour devenir quasiment nulle en janvier. Pendant les pluies, les phases larvaires deviennent plus longues, ce qui permet la survie des insectes jusqu’à la saison sèche suivante. L’existence de cycles courts de développement durant la saison sèche explique la pullulation des tabanidés à partir du milieu de cette saison (Desquesnes *et al.*, 2005).

L’abondance des différentes espèces de tabanidés varie en fonction des biotopes échantillonnés et toutes les espèces ne sont pas présentes dans les trois milieux. Le maximum de captures est réalisé en forêt secondaire, puis en milieu anthropisé et en forêt primaire.

Cette répartition peut être liée à la différenciation des paysages, la structure des milieux pouvant engendrer des microhabitats particuliers plus ou moins favorables au développement des espèces de tabanidés. En effet, la forêt primaire est caractérisée par de très grands arbres (jusqu’à 50 m de haut et plus de 2 m de diamètre) dont les cimes forment une canopée qui obscurcit le sous-bois et atténue considérablement la température; de plus, la visibilité des pièges, et donc leur efficacité, y est réduite. En revanche, la forêt secondaire est plus basse, avec une voûte interrompue; les températures y sont relativement élevées étant donné que les quantités de lumière et le temps d’éclairement y sont importants (Darchen, 1978). Dans les conditions optimales (température, humidité relative, alimentation protéique suffisante), les femelles de tabanidés sont capables de pondre une semaine après leur émergence. Ce cycle serait donc plus court dans les milieux ouverts (forêt secondaire et milieu anthropisé) qu’en forêt primaire, avec plusieurs générations qui se succéderaient, d’où des populations plus abondantes.

Les différences d’abondance peuvent également être liées aux interactions entre les effets de l’ensoleillement et la nature du piège (Doutoum *et al.*, 2002). Le pièges Vavoua et le piège N’Zi sont essentiellement constitués de tissus bleus, noirs, et moustiquaire. Doutoum *et al.* (2002) avaient utilisé le piège N’Zi et ont montré que la luminosité pourrait améliorer l’attractivité du piège, en particulier au niveau des parties bleues. Cette meilleure attractivité expliquerait les faibles captures de tabanidés obtenues dans les milieux à faible luminosité (forêt primaire) et celles assez importantes obtenues dans les milieux relativement éclairés (forêt secondaire et village).

Quant à l’indice de Margalef, qui traduit la richesse de la diversité des espèces dans le biotope, il est plus grand en forêt secondaire, puis en forêt primaire et enfin en milieu anthropisé. Les espèces les plus anthropophiles sont naturellement trouvées à proximité des habitations humaines. C’est le cas de *Chrysops silaceus* (espèce vectrice de la loase) qui n’a été rencontrée qu’au village. En revanche, *Tabanus secedens*, l’espèce la plus abondante, peut-être considérée comme une espèce de forêt puisque absente au village. Seule *Chrysops dimidiatus* (autre espèce vectrice de la loase), la troisième espèce en abondance, est rencontrée dans les trois milieux échantillonnés.

## Conclusion

Connaître les densités apparentes des mouches hématophages constitue un élément important pour la valorisation des réserves et parcs nationaux dans l’optique du développement de l’écotourisme au Gabon, car ces insectes, en particulier les *Chrysops*, sont des vecteurs de maladies humaines; il convient donc d’évaluer l’intensité des contacts hommes-insectes favorables à ces infections. De même, bien que les autres espèces de tabanidés, en particulier celles du genre *Tabanus* dont les densités sont élevées, soient des vecteurs potentiels de nombreuses maladies animales touchant notamment le bétail, certaines sont également des zoonoses (anthrax, fièvre Q, pasteurellose, listeriose, etc.). Dans les études des insectes hématophages dans la réserve de biosphère Ipassa-Makokou, notre contribution a abordé l’étude des peuplements de tabanidés. Ce travail constitue une première approche dans l’étude de cette famille, encore peu connue au Gabon. Il a permis d’identifier huit espèces appartenant à trois genres de tabanidés qui cohabitent dans les trois milieux prospectés de la réserve. Ces résultats confirment la distribution différente des genres *Tabanus* et *Chrysops* entre les milieux forestiers et le village. En effet, la répartition de ces insectes varie en fonction des biotopes explorés. En forêt primaire, les tailles de population des différentes espèces sont faibles par rapport à celles que l’on retrouve dans les deux autres milieux.

L’étude longitudinale des tabanidés se poursuit actuellement dans le parc national de l’Ivindo dans la perspective d’améliorer les résultats obtenus. Des études sur l’activité journalière et sur l’origine des repas de sang sont en cours. L’intégration de ces informations permettra d’évaluer l’importance épidémiologique de ces insectes et d’élaborer des stratégies de contrôle dans le parc national de l’Ivindo.
